# Protease-Activated Receptor-2 and Phospholipid Metabolism Analysis in Hyperuricemia-Induced Renal Injury

**DOI:** 10.1155/2023/5007488

**Published:** 2023-07-13

**Authors:** Xiaolu Sui, Tingfei Xie, Yunpeng Xu, Aisha Zhang, Yanzi Zhang, Fengjuan Gu, Lixiang Li, Zibin Xu, Jihong Chen

**Affiliations:** ^1^Department of Nephrology, The Second Affiliated Hospital of Shenzhen University, Shenzhen 518000, Guangdong, China; ^2^Department of Nephrology, The People's Hospital of Baoan Shenzhen, The Second School of Clinical Medicine, Southern Medical University, Shenzhen 518000, Guangdong, China; ^3^Department of Nephrology, Shenzhen Baoan People's Hospital (Group) The Second People's Hospital, Shenzhen 518000, Guangdong, China

## Abstract

Interstitial inflammation is an important mechanism of pathological damage in renal injury caused by hyperuricemia. Protease-activated receptor-2 (PAR2) is a class of targets that act upstream of the PI3K/AKT/NF-*κ*B pathway and is involved in various inflammatory diseases. We induced a hyperuricemia model in rats by adenine and ethambutol gavage in an *in vivo* experiment. We demonstrated that PAR2 and PI3K/AKT/NF-*κ*B pathway expression were significantly upregulated in renal tissues, with massive inflammatory cell infiltration in the renal interstitium and renal tissue injury. Treating hyperuricemic rats with AZ3451, a selective metabotropic antagonist of PAR2, we demonstrated that PAR2 antagonism inhibited the PI3K/AKT/NF-*κ*B pathway and attenuated tubular dilation and tubulointerstitial inflammatory cell infiltration. The phospholipid metabolism profiles provided a perfect separation between the normal and hyperuricemic rats. In addition, we also found that AZ3451 can affect phospholipid metabolism. Our work suggests that PAR2 may mediate hyperuricemia-mediated renal injury by activating the PI3K/AKT/NF-*κ*B pathway. The PAR2 antagonist AZ3451 may be a promising therapeutic strategy for hyperuricemia-induced inflammatory responses.

## 1. Introduction

Hyperuricemia is a metabolic disorder in which blood uric acid (UA) levels are elevated due to disorders of purine metabolism or impaired UA excretion. Hyperuricemia can lead to kidney damage, often in conjunction with chronic kidney disease, coronary artery disease, hypertension, and diabetes mellitus, and is strongly associated with poor disease prognosis [1]. Although the clinical symptoms of renal injury due to hyperuricemia are well-known, the pathophysiological and molecular mechanisms are not fully understood. The pathogenesis of renal injury due to hyperuricemia include pro/anti-inflammatory imbalance, urate deposition, oxidative stress, renin–angiotensin system activation, mesenchymal transformation of renal tubular cells, and immune disorders [2, 3]. It is widely believed that hyperuricemia induces crystal-dependent renal inflammation, with macrophages as key mediators. However, recent studies suggest that soluble UA may also have a pro-inflammatory effect, independent of crystal formation [2]. Soluble UA and UA crystals can trigger inflammation by activating NOD-like receptor protein 3 (NLRP3) inflammatory vesicles, nuclear transcription factor-*κ*B (NF-*κ*B) signaling, migration of macrophages, and other cells to the kidney for infiltration and activation, secretion of tumor necrosis factor-alpha (TNF-*α*), monocyte chemotactic protein-1 (MCP-1), IL-6, IL-18, IL-17, and other inflammatory factors and chemokines, which in turn further promote inflammatory cell infiltration, creating a waterfall effect and further amplifying the inflammatory response [2, 4–6]. Although existing studies have not fully elucidated the pathogenesis of renal inflammatory injury caused by UA, inflammation, and inflammatory cytokines certainly play a crucial role. The current standard of care for patients with hyperuricemia is UA-lowering therapy, which mainly includes xanthine oxidase (XOD) inhibitors and UA reabsorption inhibitors. In addition, some drugs targeting urate transporter 1 are still in clinical trials [7]. However, inflammation makes an important contribution to the renal injury caused by hyperuricemia, and clinical therapies targeting inflammation have not been adequately studied.

Protease-activated receptor-2 (PAR2), a G protein-coupled receptor family member, is widely expressed throughout human tissues and is activated by thrombin or tissue factor. It activates signal transduction pathways such as protein kinase C through the G protein-coupled system to produce various cytokines and inflammatory mediators, mediating the inflammatory response [8, 9]. The increasing investigation gain deeper insights into the mechanisms through which the PAR2 modulates renal disease. Activation of PAR2 on human kidney tubular epithelial cells stimulates synthesis and secretion of tissue factor that induces blood clotting [10, 11]. Besides, PAR2 exacerbate increased inflammatory cytokines in endothelial cells and podocytes in diabetic nephropathy [12]. Hayashi found that inhibition of PAR2 attenuated adenine-induced renal histological damage [13]. PAR2 induces a class of targets upstream of phosphatidylinositol 3-kinase (PI3K)/AKT/NF-*κ*B signaling that activates PI3K/AKT/NF-*κ*B signaling by altering its activity and binding to chemicals in the pericellular environment [14]. However, it is unclear whether PAR2 mediates renal injury through the PI3K/AKT/NF-*κ*B signaling pathway during hyperuricemia-induced renal injury and whether inhibition of PAR2 can protect against hyperuricemia-induced renal injury.

Our previous analysis of peripheral blood in patients with hyperuricemia and renal injury found that many lipid metabolites are potential plasma metabolic biomarkers, most of which are involved in phospholipid metabolism, suggesting that phospholipid metabolism may play a role in the pathogenesis of UA-mediated kidney injury [15]. However, a precise understanding of phospholipid metabolomics and molecular and cellular mechanisms in UA-driven renal inflammation has not been achieved. Here, we systematically evaluated the role of PAR2 in regulating the PI3K/AKT/NF-*κ*B inflammatory signaling pathway in hyperuricemia-induced renal injury. We performed adenine and ethambutol gavage in male rats to establish a model of hyperuricemia, hoping to elucidate the possible mechanisms of PAR2-mediated PI3K/AKT/NF-*κ*B signaling channels in the process of UA-induced renal damage. At the same time, we identified plasma phospholipid metabolites based on ultraperformance liquid chromatography–tandem mass spectrometry (UPLC-MS/MS).

## 2. Materials and Methods

### 2.1. Animal Models

**Figure 1:**
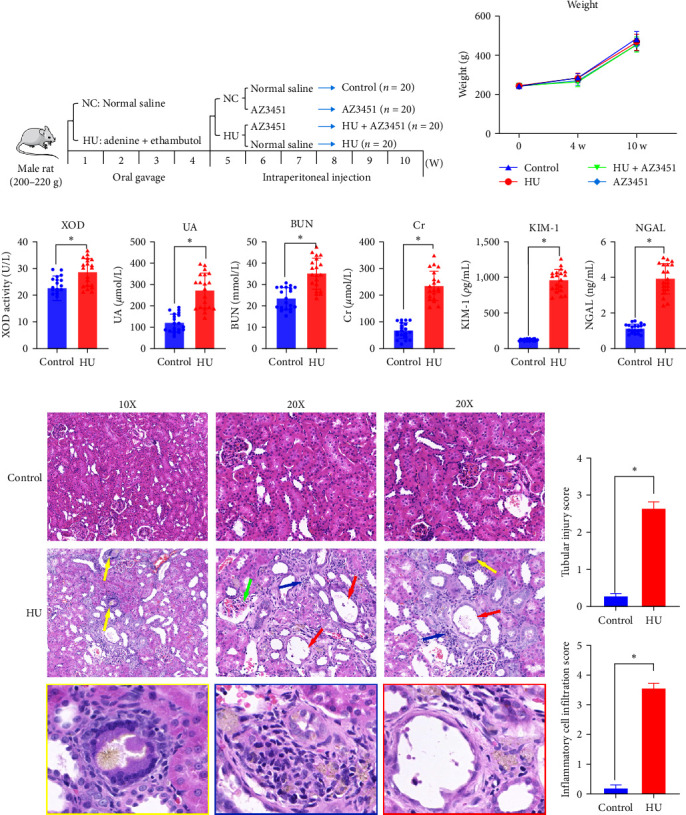
(a) Schematic representation of the experimental workflow. (b) Bodyweight of each group 0, 4, and 10 weeks after model establishment (*n* = 20). (c) Uric acid, creatinine, BUN, KIM-1, NGAL levels, and XOD activity of rat's serum after 4-week administration of adenine and ethambutol in the normal control and HU groups (*n* = 20). (d) Representative images of HE staining. The red arrows show tubular lumen dilatation and tubular epithelial cell necrosis. The yellow arrows show the brown crystals. The blue arrows show the inflammatory cells. The green arrows show glomerular damage. (e) The tubular injury and inflammatory cell infiltration score for HE staining. Data are presented as means ± standard deviation. ns, not significant.  ^*∗*^*p* < 0.05. NC, normal control; HU, hyperuricemia; XOD, xanthine oxidase; UA, uric acid; Cr, creatinine; BUN, blood urea nitrogen; NGAL, neutrophil gelatinase-associated lipocalin; KIM-1, kidney injury molecule-1.

Healthy male Sprague Dawley rats were purchased and housed in plastic cages (200–220 g). Rats were housed at a temperature of 23 ± 3°C with a relative humidity of 55% ± 15% and a 12 hr light/12 hr dark cycle. Rats were randomly divided into the normal control group and the hyperuricemia (HU) group. The rat hyperuricemia model was induced by intragastric administration of combined adenine (200 mg/kg) and ethambutol (250 mg/kg) daily for 4 weeks. The control group was given an equal amount of normal saline. Next, normal saline (control and HU groups, *n* = 20) and PAR2 antagonist AZ3451 (1 mg/kg, AZ3451 and HU + AZ3451 groups, *n* = 20) were further administered via intraperitoneal injection daily for 6 weeks (Figure 1(a)). The doses selected were based on our preliminary experiments. All procedures were carried out following the Institutional Animal Care Committee Guidelines and were approved by the Ethics Committee of the Affiliated Bao'an Hospital of Shenzhen (approval no. BYL20180208).

### 2.2. Blood and Kidney Tissue Sample Collection

Blood samples were collected after final administration at 4 weeks and final intraperitoneal injection at 6 weeks (heparin sodium anticoagulation). Serum samples were subjected to biochemical assays on the collection day. The kidneys were harvested and separated on an ice plate after the final intraperitoneal injection at 6 weeks. Parts of kidney tissues were immediately dissected and fixed in formalin for histological analysis, and the rest were stored at −80°C for further analysis. Euthanasia was performed by intraperitoneal injection of pentobarbital (100 mg/kg) at the end of the experiment.

### 2.3. Cell Culture and Transfection

Human renal proximal tubule epithelial (HK-2) cells were procured from Procell (Hubei, China) and cultured in DMEM/F12 (Hyclone Laboratories Inc, Logan, UT, USA) complemented with 10% FBS and 1% penicillin–streptomycin at 37°C in a 5% CO_2_ atmosphere. Next, 100 mL of complete medium was added to a glass flask containing 25 mg of UA powder, shaken, and heated in a water bath at 60°C. The UA concentration was determined by filtration through a sterile 0.22 *μ*m filter, and the UA working masterbatch was diluted proportionally with a complete medium to prepare a medium with a UA concentration of 720 *μ*M. Thereafter, we used this UA medium to treat HK-2 cells. siRNA against PAR2 and its negative control siRNA were synthesized in collaboration with Sangon Biotech (Shanghai, China). The sequences for si-PAR2-1 were 5′-GUG CAG AGG UAU UGG GUC ATT-3′ (sense) and 5′-UGA CCC AAU ACC UCU GCA CTT-3′ (antisense); those for si-PAR2-2 were 5′-GAA GAA GCC UUA UUG GUA ATT -3′ (sense) and 5′-UUA CCA AUA AGG CUU CUU CTT-3′ (antisense), and those for si-PAR2-3 were 5′-AAG AAA CAC UCC AGG AAA UTT -3′ (sense) and 5′-AUU UCC UGG AGU GUU UCU UTT-3′ (antisense). Thereafter, these siRNAs were transferred into HK-2 cells using Lipofectamine 2000 (Invitrogen, Waltham, MA, USA) according to the manufacturer's instructions. The CDS region of the PAR2 gene was implanted into the pCDNA3.1(+) plasmid to build the PAR2 overexpression plasmid pCDNA3.1(+)-PAR2, which was manufactured by Tianyi Huiyuan Bioscience (Hubei, China). pcDNA3.1 (+)/PAR2 was transiently transfected into HK-2 cells by polyethyleneimine.

### 2.4. Biochemical Analysis

Assay kits for UA, creatinine (Cr), XOD activity, and blood urea nitrogen (BUN) were purchased from Jiancheng Biotech (Nanjing, China). Serum UA were determined by enzymatic colorimetry. Serum Cr was determined by the sarcosine oxidase method. Serum BUN levels were determined by the urease method. Serum XOD activity was determined by colorimetry. All biochemical parameters were determined according to the manufacturer's protocol.

### 2.5. Histopathological and Immunohistochemical Analyses

The excised kidneys were fixed in 10% formalin and embedded in paraffin. Each specimen was cut into 4 *μ*m sections. Hematoxylin–eosin-stained sections were prepared to study the pathological tissues by optical microscopy. Pathological slides were scored in a blinded manner. Six samples were randomly selected from each group for pathological analysis, and the sections were digitally labeled without labeling the group and other information. Three sections from each sample were selected for scoring. Ten visual fields were randomly selected for each slice to evaluate the renal tubular injury and inflammatory cell infiltration. The tubular injury (tubular epithelial cell necrosis, absence of brush border, and tubular dilatation) was scored at 20x magnification according to the proportion of damaged tubules to total tubules as follows: 0 for no lesions, 1 for <25%, 2 for 25%–50%, 3 for 50%–75%, and 4 for >75%. The inflammatory cell infiltration was scored at 40× magnification according to the infiltration area as follows: 0 for no lesions, 1 for <10%, 2 for 11%–20%, 3 for 21%–30%, 4 for 31%–40%, and 5 for >41%. The slices were dyed using the SP method and reacted with primary antibodies against PAR2 (Abcam, Cambridge, UK; ab180953) diluted to 1 : 100 with dilution buffer. Thereafter, immunohistochemical staining with DAB and mild restaining with hematoxylin were performed. Stained images were acquired using a Nikon E100 microscope (Nikon, Tokyo, Japan).

### 2.6. Determination of PAR2, PI3K, AKT, NF-*κ*B mRNA, and Protein Levels

The RevertAid First Strand cDNA Synthesis Kit (Thermo Fisher Scientific, Waltham, MA, USA) was used to synthesize cDNA following the manufacturer's instructions. The primer sequences are shown in Supplementary 1. Real-time RT–PCR was performed using the SYBR Green PCR master mix and analyzed using a Light Cycler 480 (Roche Diagnostics GmbH, Mannheim, Germany). Proteins were separated using standard SDS-PAGE and transferred to PVDF membranes. Blots were incubated with anti-PAR2 (1 : 1,000; Proteintech, Rosemont, IL, USA; 12160-1-AP), anti-PI3K p85 (1 : 5,000 for the *in vitro* experiment; 1 : 1,000 for the *in vivo* experiment; Proteintech; 60225-1-Ig), anti-AKT3 (1 : 1,000 for the *in vitro* experiment; 1 : 2,000 for the *in vivo* experiment; Abcam; ab152157), anti-p-AKT (Ser473) (1 : 1,000, Cell Signaling Technology, Danvers, MA, USA; #4060), anti-p-NF-*κ*B p65 (Ser536) (1 : 500; Cell Signaling Technology; #3033), anti-NF-*κ*B p65 (1 : 2,000; Proteintech; 10745-1-AP). Anti-GAPDH Mouse Monoclonal Antibody (1 : 5,000; Abbkine, Wuhan, China; A01020), and anti-GAPDH Antibody (1 : 3,000; Abcam; ab37168) served as loading control substances for the *in vitro* and *in vivo* experiments, respectively. Proteins were visualized using ECL chemiluminescent substrate.

### 2.7. ELISA Assay

The cytokine concentrations (IL-6, MCP-1, TNF*α*, IL-1*β*, and TGF-*β*1) in rat kidney tissues and cell culture supernatant were measured. We determined the total protein concentration of tissues using BCA before the assay in rat kidney tissues. The cytokine concentration in rat kidney tissues was expressed using the cytokine content per gram of tissue homogenate protein. The concentration of NGAL, and KIM-1 in serum were measured. Assay kits for interleukin 6 (IL-6), monocyte chemotactic protein 1 (MCP-1), tumor necrosis factor *α* (TNF-*α*), interleukin 1*β* (IL-1*β*) (primary and mature forms), and transforming growth factor *β*1 (TGF-*β*1) were purchased from MlBio (Shanghai, China), while those for NGAL and KIM-1 were purchased from Elabscience (Wuhan, China). We measured IL-1*β* (ml037361), Pro-IL-1*β* (ml027415), TNF-*α* (ml002859), IL-6 (ml102828), MCP-1 (ml002960), TGF-*β*1 (ml002856), NGAL (E-EL- R0662c), and KIM-1 (E-EL-R3019) using the double antibody sandwich method and calculated the concentrations as per manufacturer's protocol.

### 2.8. Sample Preparation for Metabonomic Analysis

As seen in Supplementary 2, 10 internal standards were purchased from Sigma–Aldrich (St. Louis, MO, USA). The nomenclature of phospholipids is described on the LIPID MAPS website (http://www.lipidmaps.org/). Ten internal standards (100 ng of each) were added to 80 *μ*L plasma in an Eppendorf tube. Subsequently, 0.45 mL of deionized water was added to the mixture, which was then centrifuged at 12,000 rpm for 5 min at 4°C. Thereafter, the bottom layer was collected, and the top layer was subjected to the same extraction procedure. The samples collected from the two extractions were redissolved in CHCl_3_/CH_3_OH.

### 2.9. UHPLC-MS Conditions and Analysis

An Agilent 1290 ultrahigh-performance liquid chromatography system coupled with a 6,470 triple–quadrupole mass spectrometer (Agilent Technologies, Santa Clara, CA, USA) was used to analyze the complex mixture of phospholipids. A ZORBAX Eclipse Plus C18 (2.1 × 100 mm, 1.8 *μ*m; Agilent Technologies) column was used, maintaining the temperature at 50°C. The capillary voltage was set at 4.0 kV (positive ion mode) and 3.5 kV (negative ion mode). The sheath gas flow was set at 11 L/min.

All data were processed using Mass Hunter software (Agilent Technologies; B.08.00). The concentrations of phospholipid species were calculated from their relative abundances relative to the internal standard of each phospholipid class.

### 2.10. Statistical Analysis

The normality of distribution of continuous variables was tested by one-sample Kolmogorov–Smirnov test. Quantitative data were presented as means ± SD. Comparisons of two independent groups were performed by two-tailed unpaired *t*-tests or the Mann–Whitney *U* test. Intergroup differences were analyzed by one-way analysis of variance (ANOVA) followed by Dunnett's multiple comparisons test. All statistical analyses were processed using GraphPad Prism 8 (GraphPad Software, San Diego, CA, USA), and the differences were considered statistically significant when *p* < 0.05.

## 3. Results

### 3.1. Administered Intragastrically with Combined Adenine and Ethambutol in Rats Recapitulates Hyperuricemia and Kidney Damage in Human

To investigate the mechanism of renal injury due to hyperuricemia, we established a hyperuricemia (HU) model induced by adenine and ethambutol in rats, as previously described [16] (Figure 1(a)). During the experiment, no animals died. The body weight gain of rats in the HU group was similar to that of control rats (Figure 1(b)). Adenine administered in large amounts can enhance XOD activity and increase UA production, while ethambutol can reduce UA excretion, and the combination of both can lead to a significant increase in blood UA levels in rats [17]. The baseline blood UA level in the control group rats was 110 *µ*mol/L. Compared with the control group, the XOD activity of rats in the HU group was enhanced, and the blood UA level increased approximately 2.5-fold, indicating that the adenine combined with the ethambutol gavage method successfully established a hyperuricemia model, consistent with other studies [18, 19] (Figure 1(c)). As shown in Figure 1(c), after 4 weeks of gavage with adenine combined with ethambutol, the rats showed impaired kidney function with increased serum creatinine and BUN levels. We also tested the novel kidney injury markers KIM-1 and NGAL. A gradual decrease in serum KIM-1 and NGAL levels was seen with the cessation of adenine and ethambutol administration. In addition, HE staining showed varying degrees of degeneration and necrosis of renal tubular epithelial cells, dilatation of the tubular lumen, brown needlelike crystals of various sizes in some of the tubules, and a large number of inflammatory cells infiltrating the tubular interstitium (Figures 1(d) and 1(e)).

### 3.2. The PI3K/AKT/NF-*κ*B Pathway is Highly Upregulated in Hyperuricemia Rat Renal Tissue

**Figure 2:**
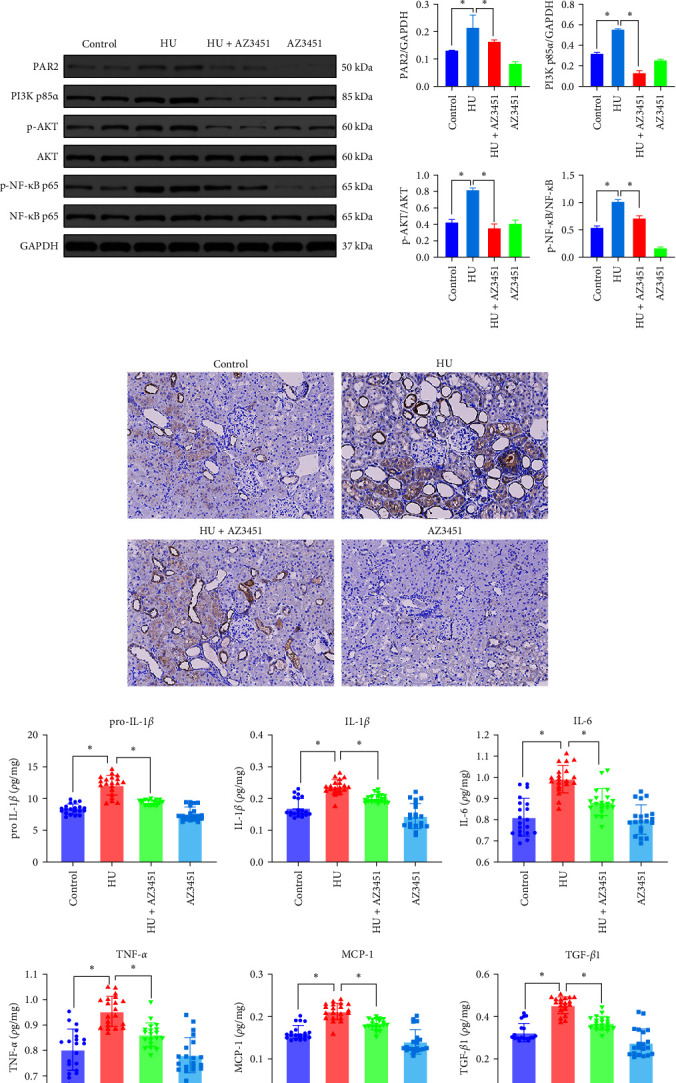
The expression of PI3K/AKT/NF-*κ*B signaling pathway genes, protein, and downstream factors in rat renal tissue. (a) Expression of PAR2, PI3K, AKT, and NF-*κ*B genes was examined by RT–PCR (*n* = 20). (b) Expression of PAR2, PI3K, AKT, and NF-*κ*B proteins were examined by Western blotting (*n* = 20). (c) PAR2 localization was examined in renal sections using immunohistochemistry. (d) The levels of pro-IL-1*β*, IL-1*β*, IL-6, MCP-1, TNF-*α*, and TGF-*β* in rat kidneys were examined by ELISA assay (*n* = 20). Data are presented as means ± standard deviation. *n* = 20 per group; ns, not significant.  ^*∗*^*p* < 0.05. Ade, adenine; Eth, ethambutol; TGF-*β*1, transforming growth factor-*β*1; TNF-*α*, tumor necrosis factor-*α*; IL-6, Interleukin-6; pro-IL-1*β*, pro-inflammatory cytokine interleukin-1*β*; IL-1*β*, interleukin-1*β*; MCP-1, monocyte chemotactic protein-1.

The PI3K/AKT/NF-*κ*B pathway plays a key role in cell metabolism, function, death, and survival. In our model, the gene and protein expression of PAR2 was significantly upregulated in the kidney tissues of HU rats (treated with adenine and ethambutol) compared with those of controls (Figure 2(a)–2(c)), and subsequently, changes in the PI3K/AKT/NF-*κ*B signaling pathway were detected. RT–PCR results showed that PI3K, AKT, and NF-*κ*B gene expression was upregulated in the kidney tissues of hyperuricemic rats compared with those of controls (Figure 2(a)). Western blotting showed a significant increase in PI3K expression and phosphorylation of AKT and NF-*κ*B p65 in the kidney tissues of hyperuricemic rats relative to those of controls (Figure 2(b)). In addition, ELISA revealed that TNF-*α*, MCP-1, IL-6, Pro-IL-1*β*, IL-1*β*, and TGF-*β*1 levels were elevated in the kidney tissues of hyperuricemic rats compared with those of controls (Figure 2(c)).

Furthermore, we treated HU rats with the PAR2 selective antagonist AZ3451 (HU + AZ3451 group), which binds to a distal metastable site outside the helical bundle of PAR2 and prevents the structural rearrangement required for receptor activation and signaling [20, 21]. We found that AZ3451 inhibited the phosphorylation of PI3K, AKT, and NF-*κ*B and reduces the production of inflammatory factors, but does not affect the mRNA levels of PI3K, AKT, and NF-*κ*B. (Figures 2(b) and 2(d)). We thought it was because antagonist binding prevented the structural rearrangement required for receptor activation and signaling, but did not affect gene expression downstream of signaling [20]. This observation suggests that PAR2 is an important sensor driving the hyperuricemia-mediated inflammatory response.

### 3.3. PAR2 Antagonist AZ3451 Attenuates Hyperuricemia-Induced Renal Injury

**Figure 3:**
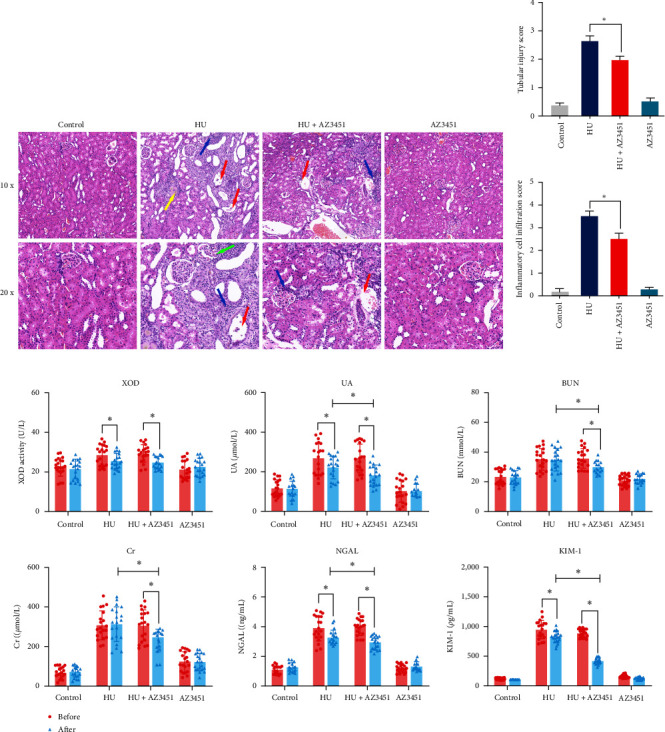
The effects on renal injury in hyperuricemia rats induced by PAR2 antagonist AZ3451. (a) Representative images of HE staining. The red arrows show tubular lumen dilatation and tubular epithelial cell necrosis. The yellow arrows show the brown crystals. The blue arrows show the inflammatory cells. The green arrows show glomerular damage. (b) The tubular injury and inflammatory cell infiltration score for HE staining. (c) The level of XOD activity, UA, Cr, BUN, KIM-1, and NGAL in rat serum. Before: before intraperitoneal administration of the control reagent or AZ3451. After 6 weeks of intraperitoneal administration of the control reagent or AZ3451. Data are presented as means ± standard deviation. *n* = 20 per group; ns, not significant.  ^*∗*^*p* < 0.05. HU, hyperuricemia; XOD, xanthine oxidase; UA, uric acid; Cr, creatinine; BUN, blood urea nitrogen; NGAL, neutrophil gelatinase-associated lipocalin; KIM-1, kidney injury molecule-1.

Given that PAR2 is upregulated in the renal tissues of hyperuricemic rats and is associated with renal inflammation and renal tissue injury, inhibition of PAR2 inhibits PI3K/AKT/NF-*κ*B pathway activation. We further explored whether PAR2 antagonists could ameliorate renal injury caused by hyperuricemia *in vivo*. As shown in Figure 3(a), we determined that AZ3451 intraperitoneal injection effectively attenuated hyperuricemia-induced renal tubular dilatation as well as tubulointerstitial inflammatory cell infiltration (Figure 3(a)). The semiquantitative scoring of renal tubular injury and inflammatory cell infiltration indicated that tubular injury amelioration in HU rats was improved after AZ3451 treatment (Figure 3(b)). After 6 weeks of intraperitoneal administration of the control reagent in the HU group, a gradual decrease in XOD activity and UA levels was observed with the cessation of adenine and ethambutol administration. However, no significant decrease in serum Cr and BUN levels were observed in the HU group. But we observed significantly reduced serum Cr and BUN levels in the HU + AZ3451 group compared with those in HU rats (Figure 3(c)). After 6 weeks of intraperitoneal AZ3451 administration, we also observed significantly reduced serum KIM-1 and NGAL levels in the HU + AZ3451 group compared with those in the HU group (Figure 3(c)). The data showed that PAR2 inhibition significantly alleviated renal injury and interstitial inflammatory response.

### 3.4. PI3K/AKT/NF-*κ*B Pathway Is Highly Upregulated in Uric Acid-Treated HK-2 Cells

**Figure 4:**
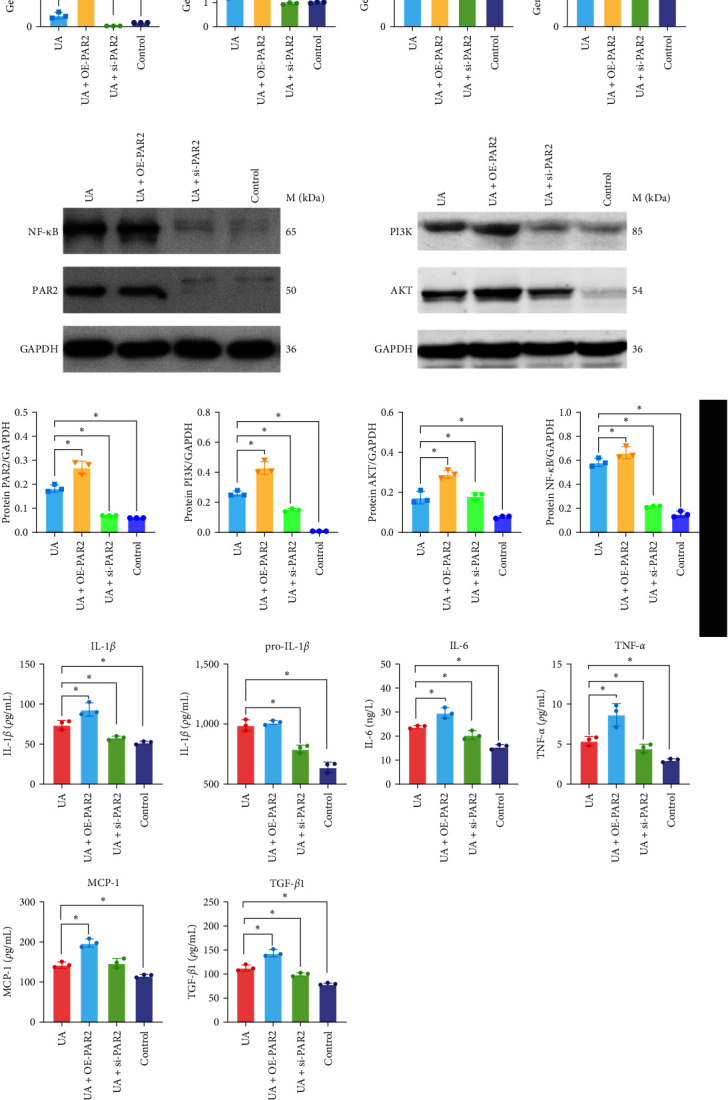
The effects on PI3K/AKT/NF-*κ*B pathway in HK-2 cells induced by uric acid (720 *μ*M). HK-2 cells were treated with pCDNA-PAR2 and siPAR2. (a) RT–PCR examined the expression of PAR2, PI3K, AKT, and NF-*κ*B genes. (b) Western blotting determined the expression of PAR2, PI3K, AKT, and NF-*κ*B proteins. (c) Quantitative values of the relative expression levels of PAR2, PI3K, AKT, and NF-*κ*B. (d) The levels of pro-IL-1*β*, IL-1*β*, IL-6, MCP-1, TNF-*α*, and TGF-*β* in the cell culture supernatant were determined by ELISA assay. Data are presented as means ± standard deviation. *n* = 3 per group; ns, not significant.  ^*∗*^*p* < 0.05.

We performed *in vitro* experiments and yielded comparable results to those of HK-2 cells treated with UA to verify the above results. Western blotting verified successful knockdown and overexpression of PAR2 (Supplementary 3). Compared with the control group, transcriptional upregulation of PAR2, PI3K, AKT, and NF-*κ*B genes and significantly increased protein expression levels confirmed that UA could activate the PAR2 and PI3K/AKT/NF-*κ*B pathway. After overexpress of PAR2, we observed further upregulation of PAR2, PI3K, AKT, and NF-*κ*B mRNAs and proteins in HK-2 cells. In addition, we synthesized siRNA against PAR2 to knock down PAR2; the combination of UA plus si-PAR2 significantly downregulated PI3K, AKT, and NF-*κ*B gene transcripts and protein expression, and significantly decreased inflammatory cytokine levels compared with those of the hyperuricemia group (Figure 4(a)–4(d)). The above data confirm that intervention with PAR2 affects UA-mediated activation of the PI3K/AKT/NF-*κ*B signaling pathway.

### 3.5. Analysis of Plasma Phospholipid Metabolism in Rats with Hyperuricemia

**Figure 5:**
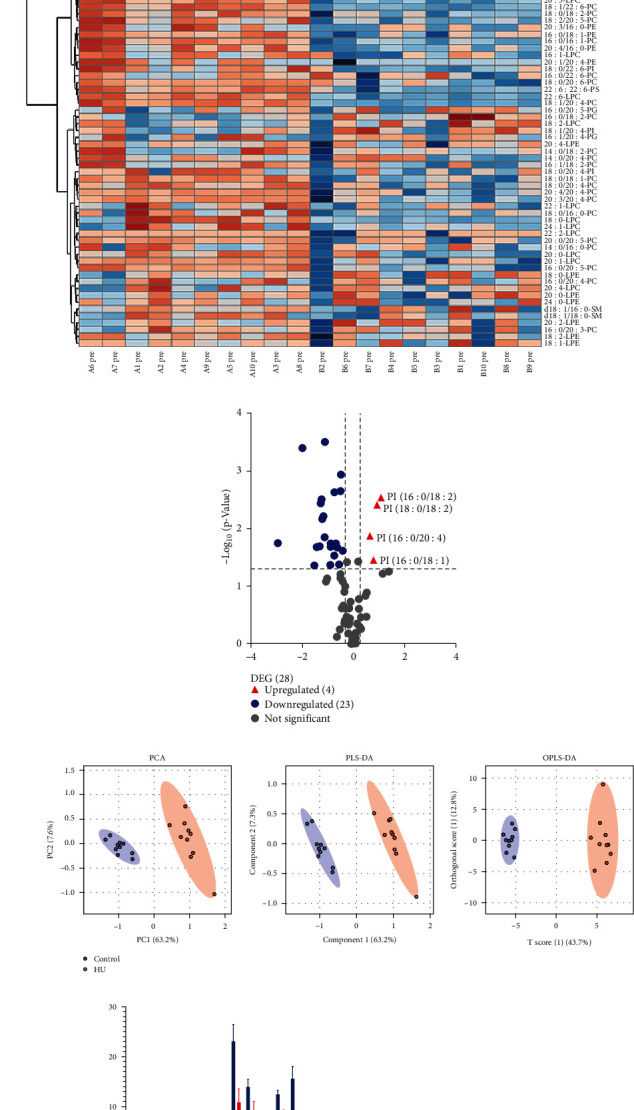
Analysis of rat plasma phospholipid metabolism. (a) Heatmap of differential metabolites between the HU and control groups. (b) The volcano map of statistically significant phospholipid metabolites. Compared with the control group, a *p*-value of less than 0.05 and multiple changes greater than 1.2-fold or less than 0.8 in the univariate analysis indicate statistical significance (*n* = 10). (c) PCA, PLS-DA, and OPLS-DA score plot (*n* = 10). (d) The concentration of plasma phospholipid in control and HU rats that *p* < 0.05. Data are presented as means ± standard deviation; *n* = 10. Blue indicates the control group; red indicates the HU group.

To gain more insight into the response in rats following hyperuricemia, we performed a phospholipid analysis to compare the phospholipid molecular content in the hyperuricemia and control groups. Phospholipid species were quantified using relative response and internal standards. Through database searching, a total of 10 types of phospholipids and 83 types of phospholipid molecules were identified in rat plasma; 78 types of phospholipid molecules were quantified (Figure 5(a)). A *t*-test or Mann–Whitney *U* test was performed on 78 phospholipid metabolites, and statistical analysis revealed that 30 phospholipid metabolites had a *p*-value of less than 0.05. The volcano graph analysis shows 27 phospholipid metabolites with a *p*-value of less than 0.05 and multiple changes greater than 1.2-fold or less than 0.8 in univariate analysis. The difference between the 27 phospholipid metabolites was statistically significant (Figure 5(b)).

At the same time, the detected phospholipid metabolites were analyzed by multivariate statistics. According to the PCA and OPLS-DA model score charts, there was a clear separation trend between the two groups, indicating that the difference between the two groups of phospholipid metabolites was statistically significant (Figure 5(c)). In the hyperuricemia group, the levels of PI (16 : 0/18 : 2), PI (16 : 0/18 : 1), PI (16 : 0/20 : 4), and PI (18 : 0/18 : 2) were significantly increased, whereas those of LPC (20 : 5), LPC (20 : 3), LPC (20 : 2), LPC (22 : 6), PC (16 : 0/16 : 1), PC (16 : 0/20 : 5), PC (18 : 1/18 : 2), PC (18 : 0/18 : 1), PC (18 : 2/20 : 5), PC (18 : 1/20 : 4), PC (18 : 0/20 : 3), PC (20 : 0/18 : 2), PC (18 : 1/22 : 6), PC (20 : 2/20 : 4), PC (20 : 0/20 : 5), PC (22 : 6/20 : 4), LPE (16 : 1), LPE (20 : 3), PE (16 : 0/18 : 1), PE (18 : 0/18 : 2), PE (18 : 0/20 : 4), PE (20 : 1/20 : 4), and PS (22 : 6:22 : 6) were significantly decreased compared with those in the control group (Figure 5(d)).

To analyze the effect of PAR2 antagonist (AZ3451) on phospholipid metabolism, we performed a phospholipid analysis to compare the hyperuricemia group with the HU + AZ3451 group at 6 weeks post-AZ3451 intraperitoneal injection. Compared with the hyperuricemia group, the levels of LPC (14 : 0), LPC (20 : 5), LPC (20 : 3), LPC (22 : 6), and PC (18 : 0/20 : 3) were significantly increased in the HU + AZ3451 group (Table 1).

## 4. Discussion

It is generally accepted that inflammation, apoptosis, and autophagy are involved in hyperuricemia-mediated renal injury [2]. However, there is still a lack of knowledge on the molecular mechanisms and therapeutic targets of inflammation in hyperuricemia-induced renal injury.

We induced hyperuricemia using adenine and ethambutol gavage, which also induces renal tubular interstitial inflammation and tubulointerstitial fibrosis, as previously described [17]. We demonstrated that PAR2 and PI3K/AKT/NF-*κ*B pathway expression is upregulated in a rat model of hyperuricemia, with similar results obtained in *in vitro* experiments. We found that AZ3451 (a selective metabotropic antagonist of PAR2) inhibited hyperuricemia-mediated activation of the PI3K/AKT/NF-*κ*B pathway and attenuated the interstitial inflammatory response and renal tissue injury, which reduced the expression of inflammation-associated cytokines. Consistent with our results, adenine-treated mice were found to exhibit severe renal dysfunction, tubular atrophy, and fibrosis, with fibrin deposition, and a significant increase in tissue factor expression and PAR2 in their kidneys. Lack of PAR2 attenuated renal histological damage and reduced the expression levels of genes associated with inflammation, fibrosis, and oxidative stress [13]. Accumulating evidence emphasizes the prominent role that PAR2 plays in mediating inflammatory diseases. Despite many achievements in PAR2 pharmacology, it took nearly 25 years for the first PAR2 inhibitors to enter clinical trials, and no PAR2 antagonists have been approved for marketing [8]. To date, PAR2 antagonists include inhibitory peptides, peptidomimetics, cell-penetrable pepducins, small molecules, and antibodies. AZ3451 [22], a nonpeptide small molecule antagonist of PAR2, has been reported to significantly reduce cartilage destruction by intra-articular injection of AZ3451 [21]. Our work is a novel exploration of the PAR2 antagonist AZ3451 in mitigating renal injury caused by hyperuricemia. AZ3451 has a broader application in the treatment of inflammatory diseases.

Studies have suggested that activating the PI3K/AKT pathway has pro-inflammatory effects and mainly concentrates on the enabled effects on NF-*κ*B activity [23, 24]. Our results showed that the PI3K/AKT/NF-*κ*B pathway is activated in hyperuricemia-induced renal injury. Elevated serum urate level (hyperuricemia) is the major risk factor for monosodium urate (MSU) crystal formation [25]. Renal tubular cells stimulated by MSU crystals produce a large number of chemokines (such as CXCL-12), which result in the targeted recruitment of macrophages in the kidney [26, 27]. In addition, MSU crystals strongly activate human primary macrophages to secrete pro-inflammatory cytokines such as IL-1*β*, IL-18, and interferon through the Src/Pyk 2/PI3K signaling pathway [28]. NF-*κ*B is a key activator of inflammation and is also the premise of inflammasome activation and activates inflammatory NLRP3 by inducing pro-IL-1*β* and NLRP3 expression [29]. MSU enters the cells through endocytosis, destroys the lysosomal membrane, and releases lysosomal tissue proteases into the cytoplasm, causing NLRP3 activation [30]. Recent studies suggest that soluble UA may also have pro-inflammatory effects, independent of crystal formation. Soluble UA could also stimulate the activation of the NLRP3 inflammasome and IL-1*β* synthesis [31]. Our results showed that the activation of the PI3K/AKT/NF-*κ*B pathway in hyperuricemia-induced renal injury was blocked by AZ3451 treatment, indicating that this pathway is involved in the protective effects of AZ3451 in hyperuricemia-induced renal injury.

We used UPLC-MS/MS to quantitatively analyze phospholipids in rat plasma. There were marked differences in phospholipid subclass and molecular species composition. Twenty-seven phospholipid molecular species were differentially changed in hyperuricemic rats, particularly PI. These biomarkers can be used for screening, detecting, diagnosing, predicting, and monitoring hyperuricemia-induced renal injury, but further validation on clinical samples is needed. PI and its phosphorylated derivatives are reservoirs of intracellular messengers, which play a pivotal role in cell signal transduction, skeleton protein anchoring, and membrane protein activation. The core of this process is phosphatidylinositol diphosphate (PIP2), which can be phosphorylated by PI3K into phosphatidylinositol-3,4,5-triphosphate (PI [3–5] P3, and PIP3) [23, 32, 33]. PIP3 is a key signaling molecule for activating the PI3K/AKT pathway that recruits multiple protein kinases into the cell membrane, including protein kinases (namely, Ras superfamily 1, AKT, and BTK). The main factor downstream of AKT is NF-*κ*B, which enters the nucleus to regulate the expression of multiple inflammatory genes [34]. We found that AZ3451 can affect phospholipid metabolism, and we revealed an unexpected link between phospholipid metabolism and PAR2 in hyperuricemic rats. McHowat demonstrated that stimulation of PARs in normal human urothelial cells leading to the production of inflammatory or cytoprotective phospholipid metabolites [35]. PAR2 activates Gq and phospholipase C, illustrating the link between PAR2 and phospholipid metabolism [36, 37], but the exact mechanism involved is unclear and needs further investigation.

In summary, our results confirmed that PAR2 expression was significantly upregulated in hyperuricemia-induced renal injury. Moreover, we found that the PI3K/AKT/NF-*κ*B pathway might participate in the process of PAR2-mediated hyperuricemic renal injury. Our work provided a better understanding of the mechanism of PAR2 in hyperuricemia-induced renal injury and showed that the PAR2 antagonist AZ3451 might serve as a promising strategy for treatment. However, our approach to the study of renal injury in hyperuricemia has limitations because clinical patients are more complex than simple experimental templates, and most of them have different underlying diseases or various concomitant comorbidities. Therefore, future studies should consider complex models, such as models of chronic kidney disease with hyperuricemia or hyperuricemia with acute kidney injury, which are more appropriate for clinical patients.

## Figures and Tables

**Table 1:** Differentially changed phospholipid species in HU and HU + AZ3451 group.

No	tR (min)	MRM transitions	Metabolite identification	Fold change	Regulation	*p*
1	1.28	833.5/255	PI (16 : 0/18 : 2)	0.83	Down	0.52
2	1.45	835.5/255	PI (16 : 0/18 : 1)	0.99	Down	0.86
3	1.22	857.5/303	PI (16 : 0/20 : 4)	0.69	Down	0.90
4	1.52	861.5/283	PI (18 : 0/18 : 2)	0.94	Down	0.51
5	3.52	812/184	PC (18 : 0/20 : 3)	1.49	Up	0.05
6	0.73	568/184	LPC (22 : 6)	1.36	Up	0.05
7	0.74	546/184	LPC (20 : 3)	1.63	Up	0.05
8	0.77	542/184	LPC (20 : 5)	1.88	Up	0.04
9	0.79	468/184	LPC (14 : 0)	1.72	Up	0.03

tR, retention time, the amount of time a compound spends on the column after it has been injected. MRM, multiple reaction monitoring. MRM transitions, the precursor ions and production. Fold change, compared with the HU group, the phospholipid quantity changes in the HU + AZ3451 group.

## Data Availability

The data that support the findings of this study are available from the corresponding author upon reasonable request.

## Supplementary Materials

Supplementary Materials Supplementary 1. Primer sequences of q-PCR. Supplementary 2. Internal standards used for retention time calibration of UHPLC-MS. Supplementary 3. Expression of PAR2 proteins were examined by Western blotting.
