# Supplementary material for: Protease-Activated Receptor-2 and Phospholipid Metabolism Analysis in Hyperuricemia-Induced Renal Injury
Source: Mediators Inflamm. 2023 Jul 13;2023:5007488. doi: 10.1155/2023/5007488 (PMC10359134; doi:10.1155/2023/5007488)
Supplement: Supplementary Materials — Supplementary 1. Primer sequences of q-PCR. Supplementary 2. Internal standards used for retention time calibration of UHPLC-MS. Supplementary 3. Expression of PAR2 proteins were examined by Western blotting. [file 5007488.f1.docx]

**Tables**

**Table S1.** Primer sequences

| **Primer name** | **Primer base sequence (5' to 3')** | **Fragment (bp)** |
| --- | --- | --- |
| *F2rl1/ Par2 (rat)* | Forward: GTGGCTGCTGGGAGGTATCA  Reverse: AGGCGGCGTGTCCAATCT | 118 |
| *F2RL1/ PAR2 (homo)* | Forward: CGACCCCTTTGTCTATTACTTTG  Reverse: CTTTGAGGTGAGGGATACTTGC | 115 |
| *Pi3k (rat)* | Forward: GAAGCGTGGGGCACATGA  Reverse: CTCCCGGTAGGCACTCTGTT | 158 |
| *PI3K (homo)* | Forward: TAGTGGTGGACGGCGAAGTA  Reverse: TTGAGGGAGTCGTTGTGCTG | 151 |
| *Akt (rat)* | Forward: CGCCTGCCCTTCTACAACC  Reverse: CTTGAGCAGCCCCGAGAG | 120 |
| *AKT (homo)* | Forward: AAGGTTGGGTTCAGAAGAGGG  Reverse: AAAAGTTGTTGAGGGGATAAGG | 138 |
| *Nf-κb (rat)* | Forward: GGGGCTGACCTGAGTCTTCTG  Reverse: CCCGTTGGGGTGGTTGATA | 135 |
| *NF-κB(homo)* | Forward: TGGGGACTACGACCTGAATG  Reverse: CACGATTGTCAAAGATGGGAT | 116 |
| *Gapdh (rat)* | Forward: CAAGTTCAACGGCACAGTCAAG  Reverse: ACATACTCAGCACCAGCATCAC | 123 |
| *GAPDH (homo)* | Forward: AATCCCATCACCATCTTCC  Reverse: GGACTCCACGACGTACTCA | 81 |

**Table S2.** Internal standards used for retention time calibration

| **Internal standard** | **Precursor ion** | **Internal standard** | **Precursor ion** |
| --- | --- | --- | --- |
| LPE (13:0) | [M +H] + | LPA (17:0) | [M - H] - |
| LPC (17:0) | [M + H] + | PI (17:0/14:1) | [M - H] - |
| PE (17:0/14:1) | [M + H] + | PA (17:0/17:0) | [M - H] - |
| PC (17:0/14:1) | [M + H] + | PS (17:0/17:0) | [M - H] - |
| SM (d18:1/17:0) | [M + H] + | PG (17:0/17:0) | [M - H] - |


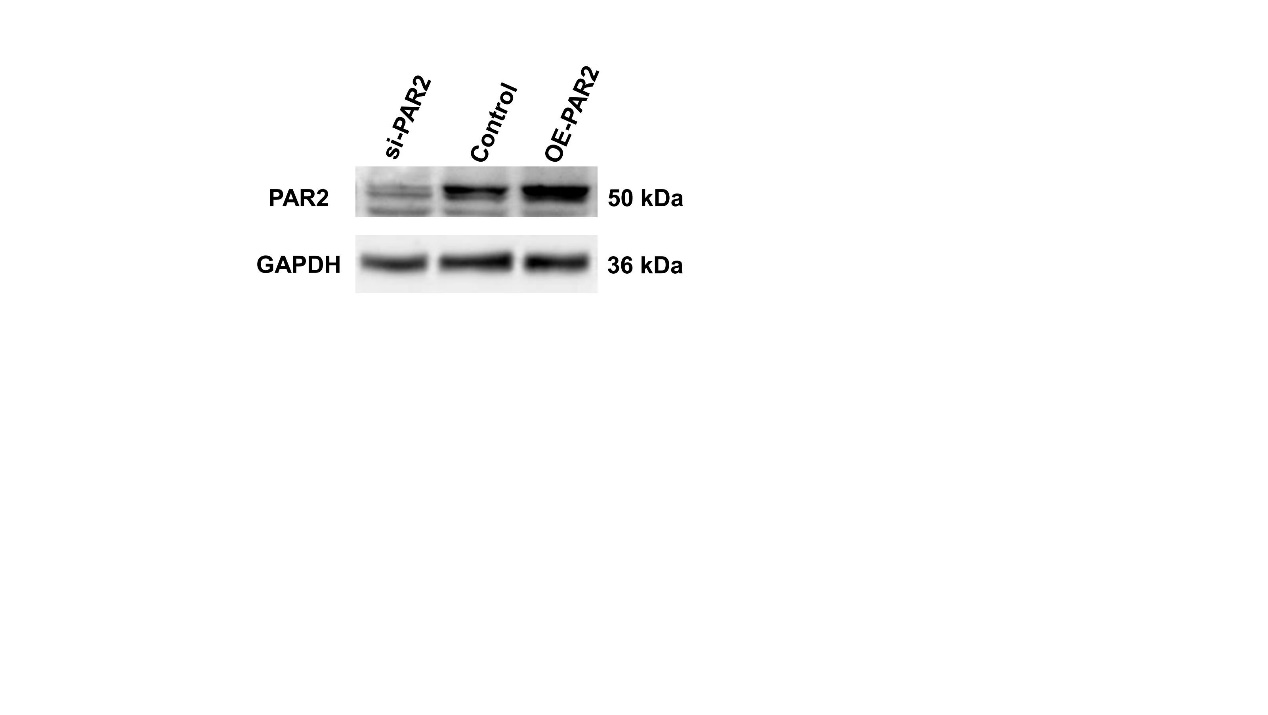


Figure S1. Western blotting determined the expression of PAR2 proteins.
